# Resistance to last-resort antibiotics in enterococci

**DOI:** 10.1093/femsre/fuaf057

**Published:** 2025-11-08

**Authors:** Zhaoxiang Lu, Ross S Mclnnes, Freya Allen, Kavita Gadar, Willem van Schaik

**Affiliations:** Institute of Microbiology and Infection and Department of Microbes, Infection and Microbiomes, School of Infection, Inflammation and Immunology, College of Medicine and Health, University of Birmingham, Birmingham B15 2TT, United Kingdom; Institute of Microbiology and Infection and Department of Microbes, Infection and Microbiomes, School of Infection, Inflammation and Immunology, College of Medicine and Health, University of Birmingham, Birmingham B15 2TT, United Kingdom; Institute of Microbiology and Infection and Department of Microbes, Infection and Microbiomes, School of Infection, Inflammation and Immunology, College of Medicine and Health, University of Birmingham, Birmingham B15 2TT, United Kingdom; Institute of Microbiology and Infection and Department of Microbes, Infection and Microbiomes, School of Infection, Inflammation and Immunology, College of Medicine and Health, University of Birmingham, Birmingham B15 2TT, United Kingdom; Institute of Microbiology and Infection and Department of Microbes, Infection and Microbiomes, School of Infection, Inflammation and Immunology, College of Medicine and Health, University of Birmingham, Birmingham B15 2TT, United Kingdom

**Keywords:** enterococcus, antibiotic resistance, antimicrobial resistance, therapeutic strategies, mechanisms of antibiotic resistance

## Abstract

The genus *Enterococcus* comprises a diverse group of species, many of which are commensal members of the gut microbiota of humans and animals. The two most prominent species associated with humans, *Enterococcus faecalis* and *Enterococcus faecium*, have also emerged as prominent opportunistic pathogens causing a range of infections in hospitalized patients, including urinary tract infections, bloodstream infections, and endocarditis. The rise of antibiotic resistance in enterococci undermines the efficacy of the treatment of infections, thus posing a significant public health risk. Enterococci readily acquire resistance to antibiotics through chromosomal mutations and the horizontal gene transfer of antibiotic resistance genes. This review offers a comprehensive examination of the mechanisms of antibiotic resistance among enterococci, with an emphasis on resistance to last-line antibiotics, including to glycopeptide antibiotics like vancomycin and teicoplanin, oxazolidinones (primarily linezolid), and daptomycin. Furthermore, we evaluate relevant candidates in the current development pipeline for antibiotics and discuss alternative strategies (phage therapy and immunotherapeutics) for the treatment and prevention of infections with multidrug-resistant enterococci. As enterococci rapidly adapt to novel conditions, including by developing resistance to new drugs and therapies, sustained research efforts are required to ensure the continuous development of treatment options for these important opportunistic pathogens.

## Teaser

In addition to having a range of intrinsic antibiotic resistance mechanisms, enterococci can efficiently acquire novel resistance mechanisms, including to last-resort antibiotics, through horizontal gene transfer, making this group of bacteria among the most problematic multidrug-resistant pathogens.

## Introduction

Enterococci are common commensals of the gastrointestinal tract of humans and animals. More than 60 species have been described in the genus *Enterococcus*, and a subset of those have been recognized as important opportunistic pathogens, causing a range of infections, particularly in immunocompromised and hospitalized patients (Lebreton et al. 2017, Fiore et al. 2019, Cattoir 2022, Schwartzman et al. 2024). These Gram-positive facultative anaerobes can thrive under harsh conditions, including high salt concentrations and a wide range of temperatures (Arias and Murray 2012). The two species that most commonly cause infections in humans are *Enterococcus faecium* and *Enterococcus faecalis*. While both species can have the capacity to evolve resistance to antibiotics through mutation and horizontal gene transfer, resistance to antibiotics appears to be spreading more rapidly in *E. faecium* than in *E. faecalis* (Zhou et al. 2020, Cattoir 2022). Beyond these two species, other species in the genus *Enterococcus* are increasingly recognized as opportunistic human pathogens (Mullally et al. 2024). Most prominently, these are *E. gallinarum* and *E. casseliflavus*, which exhibit low-level, intrinsic resistance to the antibiotic vancomycin (Yoshino 2023, Hyderi et al. 2025). In addition, sporadic clinical cases involving non-*faecalis*, non-*faecium* species—such as *E. hirae, E. durans, E. avium*, and *E. raffinosus* have been reported from bloodstream, urinary, and endocarditis infections. Infections caused with non-*faecium*, non-*faecalis* enterococci are frequently polymicrobial, with associations with Gram-negative bacilli being particularly common. Although these species are less prevalent, they can acquire high-level antibiotic resistance via mobile genetic elements (MGEs), suggesting they can serve as reservoirs of transferable multidrug resistance in healthcare settings (Eshaghi et al. 2015, Pinkes et al. 2019, Miller et al. 2020, Yoshino 2023, Mullally et al. 2024).

**Figure 1.**
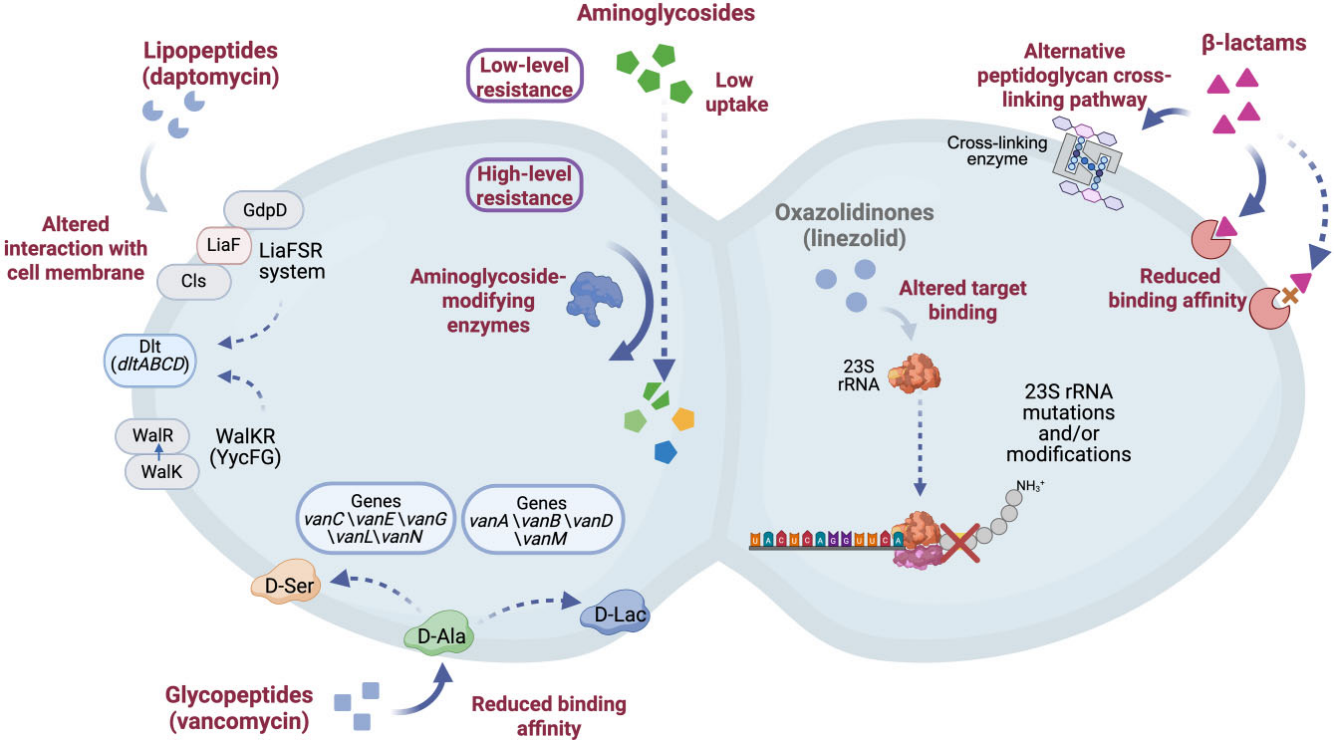
Overview of antibiotic resistance mechanisms in enterococci. Enterococci resist antibiotics through intrinsic resistance mechanisms, or after acquiring resistance, either through mutations or via horizontal gene transfer. Specifically, resistance to ampicillin in enterococci is achieved via mutations in penicillin-binding proteins that exhibit low β-lactam affinity, while intrinsic resistance is provided through the presence of alternative peptidoglycan cross-linking pathways; low-level resistance to aminoglycosides is due to barriers to the uptake of these molecules, and in the case of *E. faecium*, the chromosomal presence of a gene encoding a 6′-*N*-aminoglycoside acetyltransferase, while high-level resistance is caused by acquisition of additional enzymes that modify aminoglycosides or ribosomal mutations. Resistance to vancomycin involves changes in the peptidoglycan synthesis pathway, manifesting as reduced drug-binding affinity. Daptomycin nonsusceptibility commonly involves mutations in envelope-stress and phospholipid-metabolism pathways (e.g. LiaFSR, WalKR/YycFG, GdpD, and Cls). In addition, LiaFSR and WalKR converge on the *dltABCD* (Dlt) pathway that mediates teichoic-acid d-alanylation, increasing surface positive charge and thereby altering interactions with lipopeptides (daptomycin) and, in some contexts, glycopeptides. Linezolid resistance emerges through the acquisition of resistance genes that protect the 23S rRNA gene from the action of the antibiotic, or through mutations that affect the linezolid-binding site in the ribosome. Figure created using BioRender.com (https://BioRender.com/prhu3nq)

The formidable survival capabilities of enterococci enable them to persist in modern healthcare environments, which allows them to colonize and infect patients. In 2019, enterococci, including *E. faecalis* and *E. faecium*, were responsible for ~439 000 deaths worldwide, highlighting their significant impact as a major pathogen in clinical infections (GBD 2019 Antimicrobial Resistance Collaborators 2022). Enterococci exhibit intrinsic resistance to beta-lactams, lincosamides, and aminoglycosides, and they can readily acquire resistance to almost all antimicrobials used clinically, primarily through the acquisition of MGEs such as plasmids, transposons, and integrative elements into their genomes, but also via the mutation of antibiotic targets (Hourigan et al. 2024). Among Gram-positive bacteria, resistance to the glycopeptide antibiotics is a major concern, as these drugs are commonly used to treat infections with multidrug-resistant strains (Levine 2006). Enterococci were the first Gram-positive pathogen to acquire high-level resistance to the glycopeptide vancomycin, with the first vancomycin-resistant enterococci (VRE) being isolated in Europe in 1986 (Leclercq et al. 1988, Uttley et al. 1988). VRE has since disseminated globally as a major nosocomial pathogen (Levine 2006). Following the emergence of VRE, new antibiotics, most prominently linezolid, and daptomycin, have been developed for the treatment of infections with VRE strains. However, enterococcal strains with acquired resistance to these novel antibiotics have been reported (Bender et al. 2018). In this review, we describe the mechanisms that enterococci can acquire to gain resistance against last-line antibiotics. These diverse strategies are summarized in Fig. 1, which provides an overview of the major resistance mechanisms described to date in enterococci.

## Antibiotic resistance in enterococci

### Resistance to broad-spectrum antibiotics in enterococci

The β-lactam class of antibiotics functions by inhibiting the activity of penicillin-binding proteins (PBPs), enzymes that are essential for the synthesis of peptidoglycan, a critical structural component of the bacterial cell wall (Wacnik et al. 2022, Ren et al. 2023). This inhibition results in compromised cell wall integrity, ultimately leading to bacterial lysis and death (Lim and Strynadka 2002). Enterococci have evolved multiple intrinsic resistance mechanisms to β-lactam antibiotics, most notably through the production of PBPs that have significantly reduced affinity for these drugs. This intrinsic resistance is a key feature of enterococcal biology, which is mostly characterized in *E. faecalis* and *E. faecium*, which both express low-affinity PBPs (PBP4 and PBP5, respectively) (Zorzi et al. 1996, Rice et al. 2018, Garcia-Solache and Rice 2019, Coll et al. 2024). The low-affinity PBPs significantly reduce the bactericidal efficacy of β-lactams, making the treatment of enterococcal infections with these antibiotics less effective. In addition, these PBPs can evolve further to acquire additional mutations that lead to high-level β-lactam resistance. In addition, loss-of-function mutations in repressors of *pbp* expression can lead to overproduction of low-affinity PBPs (Singh et al. 2024, Ugalde Silva et al. 2024).

While the production of β-lactamases is the predominant β-lactam resistance mechanism in Gram-negative bacteria (Bush 2010), this mechanism seems to be of limited importance in enterococci. While *E. faecium* isolates that have acquired the staphylococcal *blaZ* gene, have been described (Zscheck and Murray 1993), these strains appear to be rare and there is no evidence that the presence of *blaZ* leads to phenotypic β-lactam resistance in *E. faecium* (Coll et al. 2024).

In addition to low-affinity PBP5, hospital-adapted *E. faecium* can resist β-lactams via an l,d-transpeptidation pathway catalysed by the l,d-transpeptidase (LDT) Ldt_fm, which serves as a bypass of PBP-mediated d,d-transpeptidation. Most β-lactams do not inhibit this route, but carbapenems can inactivate Ldt_fm (Mainardi et al. 2007, Dubee et al. 2012, Lecoq et al. 2013). However, because mutated alleles of *pbp5*, conferring resistance to β-lactams, are almost ubiquitous among clinical *E. faecium* strains (Galloway-Pena et al. 2012), the inhibition of the l,d-transpeptidation pathway by carbapenems is clinically irrelevant. In *E. faecalis*, this LDT-mediated bypass does not contribute to β-lactam resistance, with β-lactam resistance typically involving the overexpression and/or mutation of *pbp4* (Rice et al. 2018, Lazzaro et al. 2021). Compared to *E. faecium* and *E. faecalis*, resistance to β-lactams appears to be rare in other enterococci (Mullally et al. 2024).

Aminoglycoside antibiotics target the A site of the 16S rRNA fragment in the bacterial 30S ribosomal subunit, interfering with translation initiation, inhibiting peptide chain elongation, and disrupting protein synthesis (Wilson 2014). These bactericidal agents are most effective during exponential growth (Rosenberg et al. 2020). Representative aminoglycoside drugs include gentamicin, streptomycin, and tobramycin. *Enterococcus faecium* demonstrates intrinsic resistance to aminoglycosides, largely due to the chromosomal *efmM* gene, which encodes a 16S-rRNA methyltransferase that targets C1404, a key nucleotide in the drug-binding pocket, thereby lowering the activity of aminoglycosides (Costa et al. 1993, Galimand et al. 2011). High-level aminoglycoside resistance, defined as a gentamicin minimum inhibitory concentration (MIC) ≥ 500 µg/ml and streptomycin MIC ≥ 2000 µg/ml, is a horizontally acquired trait primarily mediated by aminoglycoside-modifying enzymes, which enzymatically alter aminoglycosides, rendering them ineffective (Khan et al. 2022). Aminoglycoside-modifying enzymes are classified into three main groups based on their catalytic mechanisms: *O*-phosphotransferases (APH), *N*-acetyltransferases (AAC), and *O*-nucleotidyltransferases (ANT) (Mckay et al. 1994, Wright and Serpersu 2005, Magalhaes et al. 2008, Wieninger et al. 2011, Shi and Berghuis 2012). Among enterococci, the APH enzymes encoded by the *aph(2“)-Ib* and *aph(2”)-Id* genes, along with the bifunctional enzyme AAC(6')-APH(2“), encoded by the *aac(6')-aph(2”)* gene, mediate high-level gentamicin resistance. AAC(6')-Ie-APH(2'')-Ia is the most prevalent aminoglycoside-modifying enzyme in enterococci, whereas *aph(2“)-Ib* and *aph(2”)-Id* are less commonly reported in clinical isolates (Harada et al. 2020). High-level streptomycin resistance is primarily associated with the activity of ANT genes, particularly those encoded by the *ant(6')-Ia* and *ant(3′′)-Ia* genes (Hollenbeck and Rice 2012). Many aminoglycoside resistance genes, including *aac(6')-Ie-aph(2")-Ia*, are carried by MGEs, allowing their spread among enterococci and other bacterial species (Sparo et al. 2018).

As well as the resistance genes discussed above, enterococci have also acquired resistance to other classes of antibiotics, including tetracyclines and macrolides. These resistances are largely attributed to the horizontal transfer of resistance genes, particularly *tetL* and *tetM*, which confer resistance to tetracyclines, and *ermB*, which confers resistance to macrolides (Portillo et al. 2000, Connell et al. 2003, Nishimoto et al. 2005, Wilcks et al. 2005). In addition, *E. faecium* carries the chromosomal *msrC* gene, which contributes to intrinsic macrolide resistance. *Enterococcus faecalis* lacks *msrC* and relies on acquired macrolide resistance determinants such as *erm(B)* or *mef(A/E)* (Singh et al. 2001, Werner et al. 2001, Holman et al. 2021).

### Resistance to last line antibiotics against enterococci

The diverse mechanisms of resistance to broad-spectrum antibiotics outlined above significantly complicate the treatment of enterococcal infections. For this reason, the use of the glycopeptide antibiotic vancomycin became more widespread in the 1970s and 1980s for the treatment of infections with multidrug-resistant enterococci. The increased clinical use of vancomycin, in combination with the use of the glycopeptide antibiotic avoparcin as a growth promoter in livestock production (Bager et al. 1997), acted as a strong selective pressure for the selection of resistance (Arias and Murray 2012). Vancomycin resistant isolates were first identified in the mid-1980s and have since spread globally.

### Vancomycin and other glycopeptides

Vancomycin resistance in enterococci is mediated through the acquisition of one of several gene clusters that encode enzymatic pathways, leading to the replacement of the final residue of the peptidoglycan peptide stems. These gene clusters are named *vanA, vanB, vanC*, and so on, up to *vanP*. These different gene clusters are named based on differences in their enzymatic components (Arthur and Courvalin 1993, Xavier et al. 2021). The acquisition of *vanA* and *vanB* gene clusters is the most common cause of vancomycin resistance in *E. faecium* and *E. faecalis*, while in the species *E. casseliflavus* and *E. gallinarum*, vancomycin resistance is intrinsic due to the presence of a *vanC*-type gene cluster. Occasionally, these motile species have acquired *vanA* via Tn*1546*-type elements borne on conjugative plasmids, resulting in high-level glycopeptide resistance. More rarely, *vanB* has been reported in *E. gallinarum* in association with Tn*1549*-like integrative and conjugative elements (Dutka-Malen et al. 1994, Eshaghi et al. 2015). Other gene clusters are less widespread, although the *vanM* cluster is reported to be relatively common in China, but not elsewhere (Yan et al. 2023).

**Figure 2.**
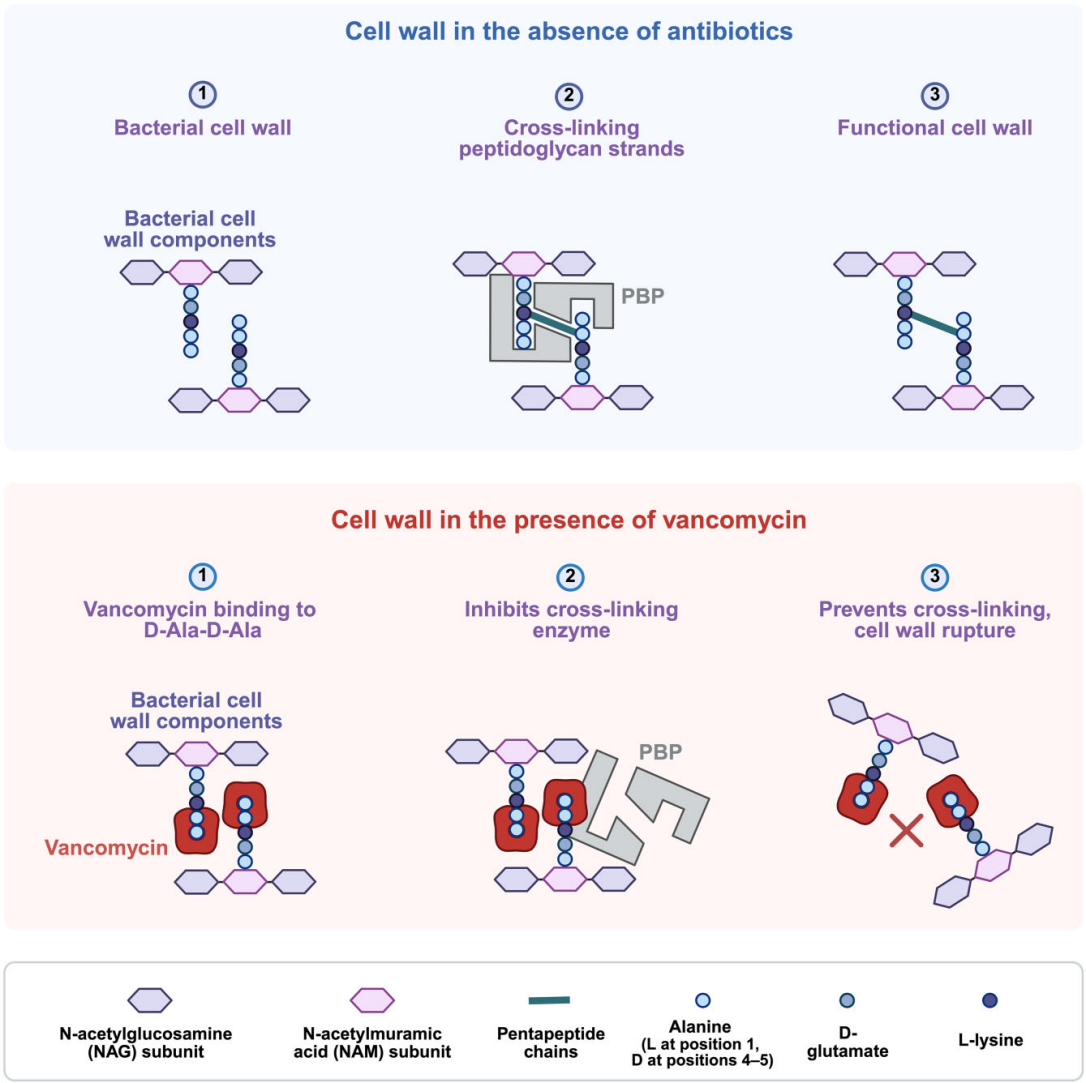
The impact of vancomycin on peptidoglycan biosynthesis. Glycopeptide antibiotics target the terminal ends of the peptidoglycan precursor, d-alanyl-d-alanine (d-Ala-d-Ala), thereby hindering the cross-linking of the peptidoglycan layers and inhibiting cell wall synthesis. Figure created with BioRender.com (https://BioRender.com/p84s430).

The *vanA, vanB, vanD*, and *vanM* gene clusters convert the terminal d-Ala residue in the peptidoglycan peptide stems to d-Lac, resulting in d-Ala-d-Lac. The affinity of vancomycin for d-Ala-d-Lac is only one-thousandth of that for d-Ala-d-Ala, hence strains with these gene clusters generally exhibit high-level resistance to vancomycin. VanP resistance has not been biochemically characterized but likely also involves the replacement of d-Ala with d-Lac (Xavier et al. 2021). On the other hand, *vanC, vanE, vanG, vanL*, and *vanN* gene clusters convert the d-Ala residue to d-Ser, resulting in d-Ala-d-Ser, which reduces the affinity of vancomycin by seven-fold, leading to relatively low-level resistance to vancomycin (Levine 2006). This alteration in the bacterial cell wall structure is critical to vancomycin’s reduced efficacy (Fig. 2). Notably, resistance conferred by *vanB* does not typically extend to teicoplanin, another glycopeptide antibiotic, which is structurally similar to vancomycin. Mechanistically, although vancomycin and teicoplanin both target d-Ala-d-Ala termini, teicoplanin does not activate the VanB two-component system (VanSB/VanRB) encoded within the *vanB* cluster; as a result, the *vanB* operon is not induced and VanB-type strains generally remain susceptible to teicoplanin. By contrast, teicoplanin efficiently activates the VanA (VanSA/VanRA) system, inducing expression of the *vanA* operon and the production of d-Ala-d-Lac termini that confer cross-resistance to both vancomycin and teicoplanin (Hill et al. 2010, Yushchuk et al. 2020). This distinction underscores the potential clinical utility of teicoplanin in infections caused by *vanB*-positive enterococci, where vancomycin therapy would be ineffective.

The dissemination of vancomycin resistance genes in enterococci is primarily driven by various MGEs, including transposons, plasmids, integrative conjugative elements, and IS elements (Paulsen et al. 2003, Wei et al. 2024). Recent studies show *vanA*/Tn*1546* often resides on Inc18-type plasmids yet can also be carried by pRUM-like (RepA_N) and linear plasmids, highlighting the diversity of plasmids that can contribute to the spread of *vanA* in enterococci (Fujiya et al. 2021, Bakthavatchalam et al. 2022, Wardal et al. 2022, Islam et al. 2023). Notably, plasmids play a crucial role in the spread of vancomycin resistance, enabling gene transfer across different bacterial species. For instance, pIP816 and high-copy-number multidrug-resistant plasmids not only carry the *vanA* gene cluster but may also harbor other resistance determinants, promoting the emergence of multidrug-resistant phenotypes in enterococci (Leclercq et al. 1988). Insertion sequence (IS) elements, such as IS*1216* and IS*1251* have been implicated in increasing *vanA* gene expression by disrupting or modifying regulatory regions within the *van* operon, particularly between *vanS*–*vanH* and *vanX*–*vanY*, which may enhance promoter activity or alter transcription termination, thereby contributing to high-level vancomycin resistance (Freitas et al. 2016).

The prevalence of vancomycin resistance is higher in *E. faecium* than in *E. faecalis* (Almeida-Santos et al. 2025). In Europe, the prevalence of vancomycin resistance among clinical *E. faecium* strains increased from 8.1% in 2012 to 19.0% in 2018, while vancomycin resistance in *E. faecalis* remained stable at 1.1% (Ayobami et al. 2020). In Asia, resistance to vancomycin appears to be more widespread, but is higher among *E. faecium* than in *E. faecalis* (22.4% versus 3.7%) (Shrestha et al. 2021). Vancomycin resistance is particularly prevalent in the USA with 82% of clinical *E. faecium* isolates having acquired resistance, which contrasts with a significantly lower prevalence (7.2%) in *E. faecalis* (Weiner-Lastinger et al. 2020). The higher prevalence of vancomycin resistance in *E. faecium* compared to *E. faecalis* is likely caused by a combination of multiple genetic and environmental factors (Davis et al. 2020, GBD 2019 Antimicrobial Resistance Collaborators 2022, Peng et al. 2022, Sannathimmappa et al. 2023). Differences in their ability to acquire and maintain MGEs play a significant role, with *E. faecium* harboring a broader and more diverse array of IS elements, such as IS*256*, IS*3*, and IS*982*, which facilitate the uptake and dissemination of resistance genes (Mikalsen et al. 2015). Additionally, the presence of IS*16*, a marker associated with hospital-adapted *E. faecium* strains, has been hypothesized to contribute to its adaptability and persistence in nosocomial environments (Leavis et al. 2007). IS*Efa5*, which has been found in multiple copies within *E. faecium* genomes, in which it undergoes frequent excision and insertion events, also contributes to genomic plasticity (Bayjanov et al. 2019). Recently, the IS*L3* family has been associated with the rapid evolution and niche adaptation of *E. faecium* in healthcare settings (Grieshop et al. 2025). The significant genomic plasticity of *E. faecium* thus enables it rapid adaptation to antibiotic selection pressures, particularly in hospital environments where horizontal gene transfer plays a pivotal role (Brodrick et al. 2016, Li et al. 2022a, Tedim et al. 2024). The fundamental mechanisms that can explain the lower prevalence of vancomycin resistance in *E. faecalis* are poorly explored but may be due to the relatively high fitness costs of the MGEs carrying vancomycin resistance genes in this species (Tedim et al. 2021).

In recent years, a subset of phenotypically vancomycin-susceptible *E. faecium* strains has been identified which carry *vanA* or *vanB* gene clusters, a phenotype known as vancomycin-variable *E. faecium* (VVE) (Hawkins et al. 2024, McInnes et al. 2024). Mechanistically, this variability often results from disruptions in the regulatory elements of the *van* operon—such as insertions of IS elements in the *vanR* gene or its promoter—which impair the inducible expression of vancomycin resistance genes (McInnes et al. 2024). VVE isolates are of clinical concern due to their potential to revert to a high-level resistant phenotype under vancomycin exposure (Thaker et al. 2015). VVE also pose a diagnostic challenge and highlight the importance of genomic surveillance to detect silent resistance determinants that may evade routine susceptibility testing (McInnes et al. 2024).

Beyond differences among *E. faecium* and *E. faecalis* in their ability to acquire and stably maintain MGEs, factors such as niche specialization, cell envelope structure, and interactions within the gut microbiome may also contribute to the differential persistence of resistance genes between *E. faecium* and *E. faecalis* (Brodrick et al. 2016, Boumasmoud et al. 2022). Further research is needed to fully elucidate the relative contributions of these factors and their impact on the epidemiology of VRE.

### Novel glycopeptide antibiotics

**Figure 3.**
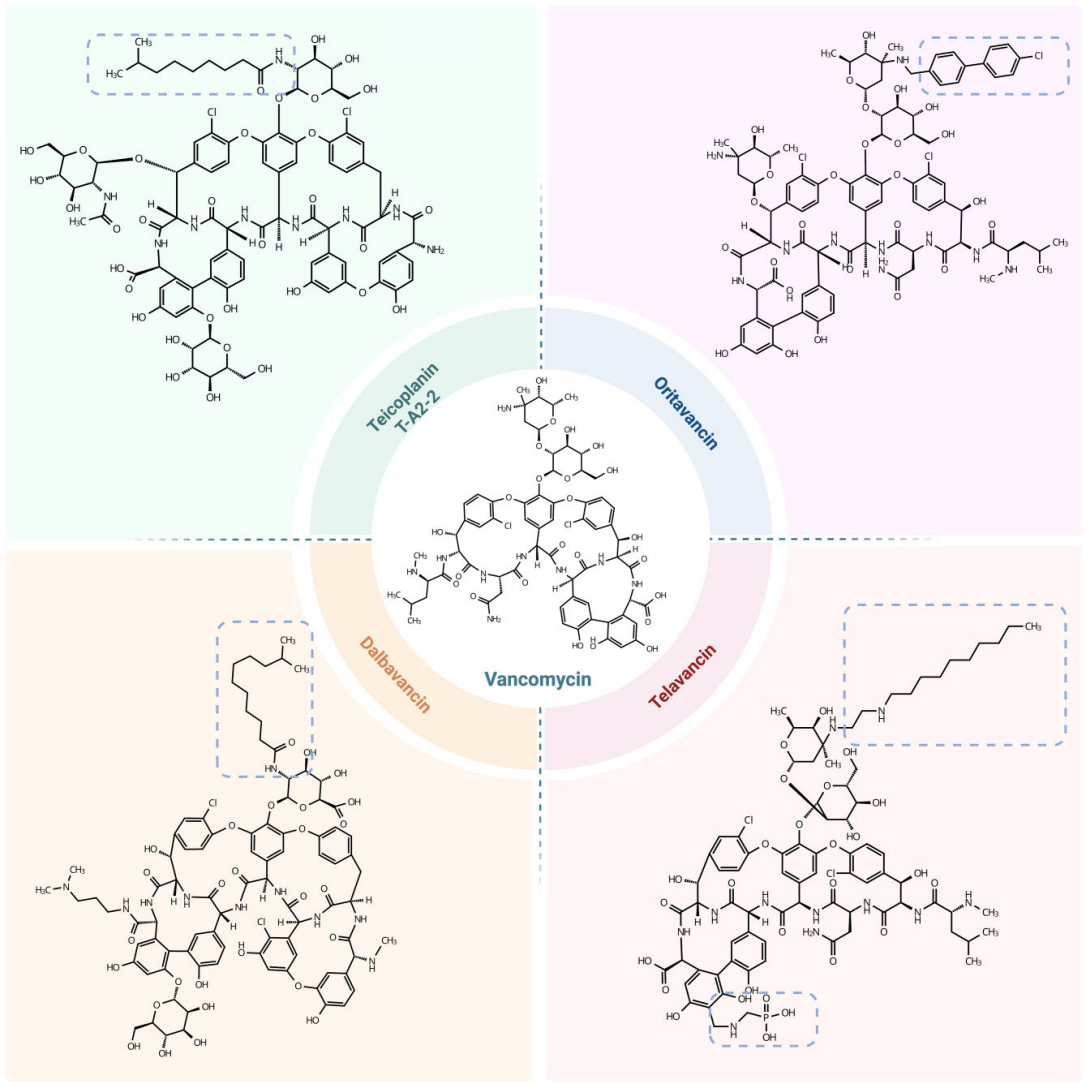
Structural comparison of glycopeptide antibiotics. Vancomycin and teicoplanin are first-generation glycopeptides with a conserved heptapeptide core, but teicoplanin features longer lipophilic side chains that enhance membrane interactions. Telavancin is distinguished by a decylaminoethyl side chain and a phosphonomethyl group, which contribute to membrane depolarization and ion leakage. Dalbavancin, derived from the teicoplanin-like compound A40926, carries an extended C11 alkyl side chain, improving pharmacokinetics and membrane affinity. Oritavancin contains a 4′-chlorobiphenylmethyl group, reinforcing membrane anchoring and enabling bacterial membrane disruption. These structural variations influence their antibacterial mechanisms and resistance profiles. Figure created with BioRender.com (https://BioRender.com/wolt2ri).

In addition to vancomycin and teicoplanin, derivatives of glycopeptides are now entering clinical practice, and these include dalbavancin, oritavancin, and telavancin (Fig. 3). These are lipoglycopeptide antibiotics, which are characterized by the addition of lipophilic side chains that enhance membrane anchoring and dimerization, leading to increased antibacterial activity and prolonged half-life (Billeter et al. 2008, Zhanel et al. 2010, Rosenthal et al. 2018). These novel antibiotics have been approved by the US Food and Drug Administration for acute bacterial skin and skin structure infections (Crotty et al. 2016, Shortridge and Flamm 2019). Dalbavancin exhibits distinct structural and pharmacokinetic advantages over traditional glycopeptides. The long hydrophobic alkyl side chain of dalbavancin reinforces membrane interactions and promotes dimerization, markedly increasing its bactericidal potency (Billeter et al. 2008). This structural modification extends the half-life of dalbavancin to ~149–250 h in humans, enabling once-weekly dosing and providing a convenient alternative to vancomycin (Billeter et al. 2008). Dalbavancin is not active against VRE with the *vanA* gene cluster, but is effective against VRE strains carrying the *vanB* gene cluster. This discrepancy may be due to the inability of dalbavancin to activate the VanR_B_/VanS_B_ two-component regulatory system that is encoded within the *vanB* gene cluster. Unlike vancomycin, which is readily sensed by VanS_B_ and triggers phosphorylation of VanR_B_ leading to induction of resistance gene expression, dalbavancin is thought to be poorly recognized by VanS. As a result, VanR_B_ is not activated and the *vanB* operon remains silent, rendering the bacteria phenotypically susceptible to dalbavancin (Hesketh et al. 2021). Although clinical resistance remains relatively rare, reports have documented reduced dalbavancin susceptibility emerging under selective pressure in other Gram-positive bacteria. In both *Staphylococcus epidermidis* and *Staphylococcus aureus*, reduced dalbavancin susceptibility has been associated with mutations in the genes encoding the WalKR two-component regulatory system, which is a key regulator of cell-wall homeostasis (Al Janabi et al. 2023). In enterococci, the WalKR (also termed YycFG/VicRK) two-component system (TCS) is highly conserved and is regarded as the only essential TCS, acting as a central coordinator of cell-envelope homeostasis. In *E. faecalis*, inhibition of transcription and translation of *walR* via an endogenous antisense RNA reduces biofilm formation and attenuates virulence *in vivo*, and experimental evolution under β-lactam pressure repeatedly selects mutations in *walKR*, consistent with a role in envelope-stress adaptation (Takada and Yoshikawa 2018, Wu et al. 2021, Zhang et al. 2023). By analogy to other species in the phylum Bacillota, specifically *S. aureus* and *Bacillus subtilis*, WalKR is expected to homeostatically control peptidoglycan hydrolases, but a direct mechanistic demonstration of this regulatory role of WalKR in enterococci is currently lacking. Thus, there is currently limited evidence for a causal relationship between mutations in *walKR* and susceptibility to dalbavancin and other glycopeptides (Ugalde Silva et al. 2024). However, as these findings suggest that WalKR may modulate glycopeptide susceptibility in enterococci, and WalKR has been proposed as a target for antibiotic development for Gram-postive bacteria (Hirakawa et al. 2020), further studies into this two-component system in enterococci are warranted.

In contrast to dalbavancin, oritavancin shows a broader spectrum of activity against VRE. Like dalbavancin, oritavancin dimerizes to enhance its binding affinity to the peptidoglycan of Gram-positive bacteria. However, oritavancin possesses additional hydrophobic modifications, including a 4′-chlorobiphenylmethyl group, which further strengthens its membrane anchoring and allows it to disrupt bacterial membrane integrity, contributing to its broader activity against VRE, including strains carrying *vanA* and *vanB* gene clusters (Belley et al. 2010). Oritavancin not only disrupts peptidoglycan synthesis via inhibition of transglycosylation and transpeptidation steps but also effectively binds to d-Ala-d-Lac, thus retaining activity against VRE (Zhanel et al. 2012, Yim et al. 2017). Notably, recent clinical studies have provided promising evidence for oritavancin’s efficacy against enterococcal infections, including those caused by VRE. A retrospective multicenter study demonstrated the successful use of oritavancin in treating bloodstream infections, endocarditis, and osteomyelitis caused by Gram-positive pathogens, including VRE (Texidor et al. 2024). Furthermore, surveillance data from European medical centres and US bloodstream infection cohorts have confirmed potent *in vitro* activity against enterococci, including strains displaying *vanA*-mediated resistance (Carvalhaes et al. 2022, Pfaller et al. 2023). Its lipophilic side chain further enables anchoring to the cell membrane, thereby enhancing its efficacy against VRE, maintaining a minimal inhibitory concentration (MIC_90_) as low as 0.06 µg/ml, even in the presence of the *vanA* gene cluster (Mendes et al. 2012). These properties position oritavancin as a promising agent for the treatment of VRE infections, warranting further clinical evaluation to define its full therapeutic potential in enterococcal disease.

Telavancin further extends the repertoire of lipoglycopeptides. Like oritavancin and dalbavancin, telavancin binds to peptidoglycan precursors to inhibit bacterial cell wall synthesis. However, it exhibits a distinct dual mechanism of action that enhances its bactericidal activity. In addition to blocking transglycosylation and transpeptidation steps by targeting lipid II, telavancin also disrupts bacterial membrane integrity. This is facilitated by its hydrophobic decylaminoethyl side chain, which interacts with the lipid bilayer, leading to membrane depolarization, ion leakage, and, ultimately, cell death (Zhanel et al. 2010). In addition to dalbavancin, oritavancin, and telavancin, next-generation vancomycin derivatives such as shapeshifting bullvalene-linked vancomycin dimers (SVDs) have been developed to address the challenge of antibiotic resistance. Unlike lipoglycopeptides, which incorporate lipophilic side chains for membrane anchoring, SVDs utilize dynamic bullvalene scaffolds to enhance dimerization and adaptability, thereby improving their efficacy against *vanA*-type VRE (Ottonello et al. 2023). Other novel glycopeptides synthesized through chemical modifications are also under investigation for their potential to overcome limitations in treating resistant strains (Belay et al. 2024).

### Oxazolidinone antibiotics and resistance

The oxazolidinone antibiotics linezolid and tedizolid were approved by the US Food and Drug Administration in 2000 and 2014, respectively, and are designed to combat infections caused by multidrug-resistant Gram-positive bacteria. These antibiotics disrupt the protein synthesis process by targeting the 50S subunit of the bacterial ribosome, inhibiting the formation of the initiation complex, similar to the mechanism of amphenicols and lincosamides (Hashemian et al. 2018, Tsai et al. 2022).

Resistance to linezolid is conferred by both chromosomal mutations and the acquisition of transferable resistance genes. Mutations within domain V of the 23S rRNA gene, specifically the G2576U mutation, significantly reduce the binding affinity of linezolid, leading to resistance (Marshall et al. 2002). Other mutations typically involve amino acid substitutions, deletions, or insertions that alter the drug-binding site in the ribosome, reducing linezolid affinity and conferring resistance (Long and Vester 2012). Beyond these chromosomal mutations, *Enterococcus* has also acquired transferable oxazolidinone resistance genes, (*cfr, optrA*, and *poxtA*), which significantly contribute to linezolid resistance (Schwarz et al. 2021).

The *cfr* gene, initially identified in *Staphylococcus* spp. from animal sources, but now found in clinical *Enterococcus* isolates, highlights the zoonotic aspect of antibiotic resistance (Tang et al. 2023). The *cfr* gene family includes multiple homologs, such as *cfr(B), cfr(C), cfr(D)*, and *cfr(E)*, which share functional similarities but differ in their sequence and distribution across bacterial species (Locke et al. 2009). Collectively, these genes encode an RNA methyltransferase that targets adenine 2503 in the 23S rRNA, leading to resistance against multiple antibiotic classes. The methylation of the A2503 residue impedes the effective binding of phenicols, lincosamides, oxazolidinones, pleuromutilins, and streptogramin A, culminating in the PhLOPS_A_ multidrug resistance phenotype (Long et al. 2006). In *Staphylococcus* and *Enterococcus, cfr* has been identified on diverse plasmids, transposons, and integrative conjugative elements, though the specific elements involved vary between genera. In *Staphylococcus, cfr* is frequently found on plasmids such as p12-00322, pSCFS3, and pSCFS7, often in association with transposons like Tn*558*, which facilitate interspecies transfer (Bender et al. 2015, Nguyen et al. 2018). In *Enterococcus*, the gene is also predominantly associated with plasmids rather than with chromosomal elements, with variations observed between *E. faecium* and *E. faecalis*. While the pE35048-oc plasmid has been reported in *E. faecium, E. faecalis* tends to harbor *cfr* on different plasmid backbones, some of which are also linked to additional resistance determinants, including *optrA* and *poxtA* (Morroni et al. 2018, Shen et al. 2023).

Studies on *Enterococcus* isolates exhibiting linezolid resistance highlighted the role of a second resistance determinant, OptrA, which influences resistance to both linezolid and tedizolid (Wang et al. 2015). Initially detected in a clinical strain of *E. faecalis*, further studies reported the presence of *optrA* in *E. faecium, E. avium, E. hirae*, and *E. gallinarum* (Nuesch-Inderbinen et al. 2022, Coccitto et al. 2023, Mullally et al. 2024). The *optrA* gene appears to be particularly promiscuous and has been reported in, other Gram-positives including *S. aureus, Streptococcus suis*, and *Streptococcus porcinus* (Wang et al. 2015, Yao et al. 2020, Xuan et al. 2023). Recent surveillance efforts in China indicate that *optrA* is predominantly detected in *Enterococcus* isolates from food-producing animals, whereas its occurrence in human isolates appears to be less frequent (Huang et al. 2022). The *optrA* gene encodes an ATP-binding cassette (ABC)-F protein, which facilitates resistance by protecting the antibiotic target site from drug binding (Sharkey and O’Neill 2018). The widespread distribution of *optrA* and its association with the use of antibiotics in livestock has raised concerns about the potential for ongoing transmission of this resistance gene among animal-associated and environmental bacteria (Tang et al. 2023).

A third linezolid resistance gene, termed *poxtA*, was initially described in methicillin-resistant *S. aureus* and its gene product was found to be an ABC-F protein, like OptrA. Amino acid identity between PoxtA and OptrA is only 32%, strongly indicating that these genes have evolved independently (Antonelli et al. 2018). Analysis of culture collections of clinical *Enterococcus* strains in different European countries found that *poxtA* and *optrA* are more common than the *cfr* genes among linezolid-resistant *Enterococcus* strains. Notably, the *optrA* gene seems to be more common in *E. faecalis* than in *E. faecium*, while the *poxtA* gene is more prevalent in *E. faecium* than in *E. faecalis* (Egan et al. 2020, Moure et al. 2020, Dejoies et al. 2021).

The dissemination of oxazolidinone resistance genes in *Enterococcus* is strongly facilitated by their association with MGEs. The *poxtA* gene is often flanked by copies of the IS*1216E*, which facilitates its mobilization and stable integration (Shan et al. 2020). Moreover, plasmids with *poxtA* frequently coharbor additional resistance determinants, such as the phenicol exporter *fexB*, and the tetracycline resistance genes *tetM* and *tetL*, suggesting that selective pressures may drive their coselection and persistence (Freitas et al. 2020, Wardal et al. 2023). Notably, experimental evidence has demonstrated that some *poxtA*-carrying plasmids possess conjugative transfer capabilities, enabling horizontal gene transfer among enterococci, further accelerating the spread of resistance (Dejoies et al. 2021, Lei et al. 2021, Shan et al. 2022). Several studies in China have shown that *cfr, optrA*, and *poxtA* are widespread in the guts of animals, and in hospital wastewater, with enterococci being the most important host of these resistance genes (Shen et al. 2024). The high prevalence in China of these resistance genes among enterococci, particularly in strains isolated in livestock, suggest that antibiotic use in agriculture poses a strong selective pressure for the emergence and spread of *optrA* and *poxtA*. The emergence and spread of plasmid-mediated oxazolidinone resistance genes is of significant public health concern. The association of these genes with enterococci from animal and environmental reservoirs strongly suggest a link to antibiotic use in agriculture. Notably, oxazolidinones are not used in livestock, but *cfr, optrA*, and *poxtA* also provide resistance to phenicol antibiotics, and this class of antibiotics is widely administered in agriculture and is regarded of limited importance for human medicine (Timmermans et al. 2021). The troubling emergence and spread of MGEs carrying oxazolidinone resistance genes may only be addressed through cross-sectoral initiatives to curb antibiotic use and an increased awareness of the risks posed by antibiotic resistance genes that provide resistance to multiple classes of antibiotics, a phenomenon termed cross-class resistance (Simjee et al. 2024).

### Daptomycin

**Figure 4.**
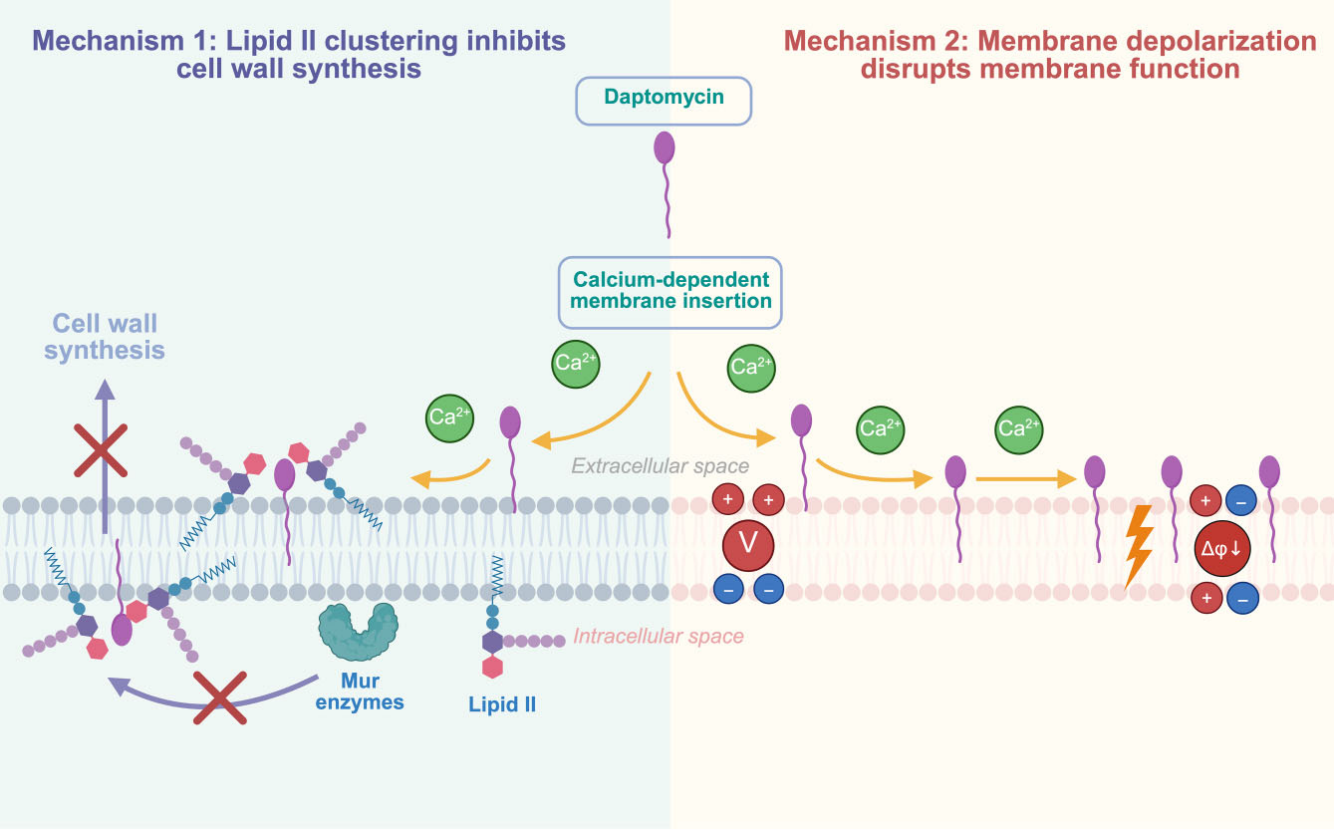
Proposed dual mechanisms of action of daptomycin. Daptomycin is a calcium-dependent lipopeptide antibiotic used against Gram-positive bacteria. Upon calcium binding, daptomycin inserts into the bacterial membrane. Left: Daptomycin induces lipid II clustering within the membrane, creating steric hindrance that prevents the membrane association of enzymes that are essential for peptidoglycan biosynthesis, primarily the lipid II synthase MurG. This spatial interference inhibits peptidoglycan synthesis and leads to a failure in maintaining cell wall growth. Right: Daptomycin disrupts membrane microdomains, leading to membrane depolarization without forming large pores. The resulting loss of membrane potential (Δψ) impairs membrane function and contributes to bacterial killing. Figure created with BioRender.com (https://BioRender.com/i9nlsl3).

Discovered in 1987 and approved for clinical use in 2003, daptomycin is a cyclic lipopeptide antibiotic that has become a vital treatment option for difficult-to-treat infections caused by Gram-positive pathogens. Unlike most membrane-targeting antibiotics that have historically been limited to topical applications, daptomycin can be administered systemically. This characteristic offers a significant advantage, as such membrane-active antibiotics tend to induce resistance more slowly compared to traditional antibiotics targeting individual protein targets (Gray and Wenzel 2020). Daptomycin has a unique mechanism as it binds to Ca^2+^ ions in a 1:1 molar ratio and these complexes insert into the cytoplasmic membrane, causing leakage of intracellular ions and adenosine triphosphate (ATP) , thus killing bacteria (Silverman et al. 2003). An alternative bactericidal mechanism has recently been demonstrated whereby daptomycin impairs cell wall synthesis by clustering lipid II in the membrane, thus preventing membrane-bound enzymes, such as MurG from accessing their substrates and halting peptidoglycan assembly (Grein et al. 2020, Buttress et al. 2024) (Fig. 4). Two factors are crucial for the antimicrobial effect of daptomycin. First, the formation of defined complexes, such as the dimeric Dap₂Ca₃PG₂ complex, enables stable and targeted binding to phosphatidylglycerol (PG)-rich domains of the bacterial membrane (Lee et al. 2017). Second, these complexes can further assemble into small, membrane-embedded oligomers that act as transient ionophores, allowing ion conduction across the membrane without forming permanent pores (Chen et al. 2014, Muller et al. 2016, Huang 2020). This localized and regulated ion leakage leads to membrane depolarization, which is one of the key events contributing to bacterial cell death. This proposed mechanism distinguishes daptomycin from classic pore-forming peptides like melittin or gramicidin and highlights a more subtle but highly specific mode of action (Huang 2020, Buttress et al. 2024).

### Mechanisms of daptomycin resistance

Concurrent with the clinical application of daptomycin, a rise in resistance among enterococci has been observed (Munoz-Price et al. 2005, Lellek et al. 2015, Chow et al. 2016, Herc et al. 2017). This resistance primarily stems from modifications in the lipid composition of the cell membrane, notably alterations in PG levels, which impair the drug’s ability to bind to the membrane. Moreover, the emergence of daptomycin resistance in enterococci appears to involve a range of mutations, particularly in genes responsible for cell membrane stress responses and phospholipid metabolism. Genes that, when mutated, are particularly commonly associated with daptomycin resistance include those in the LiaFSR system and the *yycFGHIJ* (WalKR) operon, as well as enzymes critical for phospholipid homeostasis, such as glycerophosphoryl diester phosphodiesterase (GdpD) and cardiolipin synthase (Cls) (Li et al. 2022a, Nguyen et al. 2024). Although *E. faecalis* and *E. faecium* share genetic commonalities, they exhibit distinct evolutionary trajectories to resistance upon daptomycin exposure (Miller et al. 2016), and these will be discussed further below. Among non-*faecium*, non-*faecalis* enterococci, daptomycin resistance appears to be rare, with the exception of *E. hirae* where daptomycin-resistant strains can be commonly isolated from livestock and the environment (Jackson et al. 2011, Cho et al. 2020, Mullally et al. 2024). The mechanism of daptomycin resistance in *E. hirae* is currently unknown. Below, we will also describe the recent evidence for the emergence of mobile daptomycin resistance among Gram-positive bacteria, including in *Enterococcus* (Marciniak et al. 2025).

### Daptomycin resistance in *E. faecalis*

*In vitro* evolution experiments have illuminated a common genetic pathway by which *E. faecalis* acquires daptomycin resistance. Under escalating daptomycin exposure, laboratory strains consistently develop mutations in a core set of cell-envelope regulatory and biosynthetic genes, notably those encoding the LiaFSR three-component stress response system (Arias et al. 2011). Notably, these same mutations have also been identified in clinical isolates, underscoring a convergent evolution of resistance mechanisms *in vitro* and *in vivo* (Kristich et al. 2014). Cumulative evidence suggests an ordered, two-step progression: an initial mutation in the LiaFSR regulator (such as a truncation in *liaF* or a point mutation in *liaR*) that activates the cell-envelope stress response, followed by alterations in membrane phospholipid metabolism (via *cls, gdpD*, or related genes) that remodel the cell membrane and reduce daptomycin’s binding efficacy (Arias et al. 2011). In addition to these canonical resistance mutations, other genetic contributors have been documented. For example, one *in vitro* study identified mutations in seven loci, including a global transcriptional regulator (*rpoN*), during stepwise daptomycin adaptation, hinting at alternate routes to resistance when the primary pathway is blocked (Kristich et al. 2014). Likewise, transposon sequencing (Tn-seq) approaches have reinforced the centrality of the LiaFSR circuit and membrane homeostasis genes: disrupting the LiaFSR-associated factor *liaX* or cell-envelope remodeling pathways (e.g. the *dlt* operon responsible for teichoic acid d-alanylation) sensitizes *E. faecalis* to daptomycin, confirming these elements as crucial for survival under antibiotic stress (Gilmore et al. 2020).

Uniquely, *E. faecalis* shows a reorganization of phospholipid microdomains within the cell membrane, distinct from patterns observed in *S. aureus* or *E. faecium*. In nonresistant strains, anionic phospholipids such as cardiolipin are densely packed at the division septum and polar areas. Although PG is the primary binding target of daptomycin, cardiolipin plays an essential role in organizing membrane curvature and domain localization, particularly at sites of cell division. In resistant strains, these structures are disrupted, leading to the redistribution of these lipids, thereby blocking daptomycin’s access to its target sites, effectively reducing its bactericidal activity (Tran et al. 2013). This rearrangement of phospholipids is thought to act as a decoy mechanism to divert the antibiotic from essential membrane areas.

The observed lipid redistribution may represent a downstream effect of regulatory reprogramming mediated by the LiaFSR system. In particular, mutations such as D191N in LiaR mimic phosphorylation and induce a conformational shift from dimer to tetramer, greatly enhancing DNA-binding affinity and upregulating the stress response (Davlieva et al. 2015). Deletion of *liaR*, on the other hand, results in a hypersusceptible phenotype, with MICs dropping to as low as 0.094 µg/ml, highlighting the system’s pivotal role in mediating daptomycin resistance (Reyes et al. 2015).

Mutations in the LiaFSR pathway are often accompanied by additional genetic changes that contribute to a more robust daptomycin-resistant phenotype in *E. faecalis* (Nguyen et al. 2024). For example, alterations in the *cls* gene encoding cardiolipin synthase—such as the R218Q substitution or the N77–Q79 deletion—enhance resistance in laboratory strains like OG1RF (Palmer et al. 2011). Similarly, mutations in *gdpD* have been shown to synergize with *liaF* mutations, resulting in a fully resistant phenotype​ (Arias et al. 2011). Additional mutations have been identified in the *liaXYZ* operon, which is transcriptionally regulated by LiaR. These genes are thought to modulate membrane remodeling in response to envelope stress. Notably, LiaY colocalizes with Cls and contributes to the formation of nonseptal cardiolipin-enriched microdomains, a key feature of daptomycin resistance (Miller et al. 2013). Furthermore, mutations in *yybT* and *gshF*, which are associated with cyclic dinucleotide signaling and glutathione metabolism respectively, have been identified in resistant strains. Although their precise roles remain under investigation, these genes are implicated in broader stress–response networks that may reinforce cell envelope resilience during antibiotic challenge (Miller et al. 2013).

### Daptomycin resistance in *E. faecium*

As with *E. faecalis*, daptomycin resistance in *E. faecium* arises through multifaceted genetic and biochemical adaptations of the cell envelope. While there are functional parallels to daptomycin resistance in *E. faecalis, E. faecium* also evolves daptomycin resistance through unique pathways. Prominent mutational pathways to resistance involve the LiaFSR three-component regulatory system and the essential WalKR two-component system, a conserved regulator of cell-envelope stress responses in Gram-positive bacteria (Davlieva et al. 2013, Zeng et al. 2022, Turner et al. 2024). Constitutive activation of LiaFSR, as seen in daptomycin-resistant isolates, triggers broad transcriptional reprogramming that leads to a fortification of the cell envelope—for example, through the increased expression of the *dltABCD* operon responsible for d-alanylation of lipoteichoic acids and other membrane stress defenses, which may reduce the antibiotic’s ability to reach, bind to and disrupt the cytoplasmic membrane (Zeng et al. 2022). Notably, recurrent mutations such as W73C in LiaR and T120A in LiaS have been reported in clinical isolates, and appear to coevolve to enhance resistance (Diaz et al. 2014). Alterations in the WalKR two-component system have been shown in daptomycin-resistant *E. faecalis* to lead to a remodeling of peptidoglycan synthesis and septal division, producing a thicker cell wall that reduces the lytic efficacy of daptomycin (Nguyen et al. 2022). The impact of *walKR* mutations on *E. faecium* remains to be elucidated.

Another frequent adaptation is mutation of the *cls* gene, which leads to a perturbation of the membrane phospholipid composition; *cls* mutations redistribute anionic cardiolipin and decrease the net negative charge on the cell surface, impeding daptomycin’s calcium-dependent binding to the bacterial membrane (Li et al. 2022b). Consistent with these genetic changes, daptomycin-resistant *E. faecium* exhibit altered membrane physiology, including a more positively charged cell envelope and reduced membrane fluidity, which is achieved through phospholipid remodeling, such as increased lysyl-PG and cyclopropane-modified fatty acids (Nguyen et al. 2022). While these modifications enhance survival under daptomycin pressure, they often incur fitness trade-offs: resistant mutants tend to grow slower, have attenuated virulence, and compete poorly in the absence of the drug. In some cases, the envelope changes even confer collateral sensitivities—for instance, certain daptomycin-resistant *E. faecium* become resensitized to glycopeptide antibiotics due to linked alterations in cell-envelope regulation (Zeng et al. 2022). Interestingly, in the absence of antibiotic pressure, insertion of IS elements into the *liaFSR* locus has been observed, reversing resistance and restoring susceptibility (Sinel et al. 2016).

Notably, recent findings revealed the emergence of dominant hospital-adapted *E. faecium* lineages that combine daptomycin resistance with bacteriocin production, allowing them to outcompete other enterococcal strains in the gut and thrive despite the fitness costs of resistance (Mills et al. 2025). Further novel insights into the evolution of daptomycin resistance in *E. faecium* were revealed by the observation that rifaximin prophylaxis can contribute to the emergence of daptomycin resistance in *E. faecium*. Mechanistically, rifaximin selects for mutations in the *rpoB* gene (encoding the β subunit of RNA polymerase), which leads to the increased expression of the *prdRAB* operon and membrane remodeling, conferring resistance to daptomycin (Turner et al. 2024). Taken together, current evidence indicates that daptomycin resistance in *E. faecium* is not the result of a single mutation but rather a coordinated suite of regulatory and membrane adaptations, chiefly involving LiaFSR/WalKR signaling and membrane lipid remodeling, that bolster the cell envelope against the antibiotic, albeit often at the expense of overall fitness.

### Emergence of a mobile daptomycin resistance mechanism

Until recently, all characterized mechanisms of daptomycin resistance in enterococci were thought to arise through chromosomal mutations. However, a recent study identified a novel horizontally acquired resistance determinant, the *drc* gene cluster, in a livestock-associated *Mammaliicoccus sciuri* isolate (Marciniak et al. 2025). This mobile locus encodes a BceAB-like ABC transporter and its regulatory system, which together mediate high-level daptomycin resistance by chemically inactivating the antibiotic (Kirchner et al. 2024). Unlike typical chromosomal mutations in genes, such as *walKR* or *liaFSR*, this mechanism represents a distinct strategy, expanding the known repertoire of daptomycin resistance in Gram-positive bacteria.

Of particular concern, the *drc* locus has also been identified in three *E. faecalis* isolates of animal origin, one of which exhibited phenotypic daptomycin resistance. In these strains, *drc* was flanked by IS elements or Tn3-like recombinases, and in at least one case appeared to reside on a plasmid, strongly suggesting its mobility within enterococcal populations (Marciniak et al. 2025). While its prevalence remains low and largely confined to veterinary settings, the potential for transmission into human-associated enterococci raises significant clinical concerns. Continued genomic surveillance and strict antimicrobial stewardship will be essential to limit the emergence and spread of this transferable resistance element.

## Future perspectives and concluding remarks

The issue of multidrug resistance in *Enterococcus*, especially *E. faecium*, is increasingly problematic, as this pathogen adeptly accumulates antibiotic resistance genes via horizontal gene transfer. To combat the rising tide of antibiotic resistance among enterococci, there is a crucial need for innovative treatments beyond traditional antibiotics.

The therapeutic landscape against VRE is evolving beyond conventional antibiotics, incorporating both next-generation synthetic agents and innovative biologics. Delpazolid, a next-generation oxazolidinone that inhibits protein synthesis via the 50S ribosomal subunit, has shown reduced hematologic toxicity in Phase 2 trials (Cairns et al. 2023). Iclaprim, a diaminopyrimidine targeting bacterial dihydrofolate reductase, has completed Phase 3 studies and exhibits potent anti-VRE activity in preclinical models (Sader et al. 2009). Among biologic strategies, an oral bacteriophage cocktail (VRELysin) is under Phase 1/2 evaluation for selective intestinal VRE decolonization. In parallel, engineered phage-derived lysins—cell wall-degrading enzymes—have demonstrated potent bactericidal activity against refractory *E. faecium* and *E. faecalis* isolates in preclinical models (Cairns et al. 2023, Fujimoto et al. 2024). Monoclonal antibodies targeting *Enterococcus* surface antigens, such as capsular polysaccharides and secreted virulence factors, have shown enhanced opsonophagocytic killing *in vitro* and are advancing through late preclinical development (Kalfopoulou et al. 2019). While most of these strategies remain investigational, they collectively expand the future therapeutic arsenal and underscore the urgency for translational research focused specifically on multidrug-resistant *Enterococcus* (Cairns et al. 2023).

Beyond these investigational strategies, a number of additional agents with promising antienterococcal activity have emerged from the antibiotic development pipeline. These include compounds with novel mechanisms of action or improved pharmacological properties. Radezolid, a second-generation oxazolidinone featuring a biaryl spacer and a heteroaryl side chain that improves ionization and hydrophilicity at physiological pH, has demonstrated superior *in vitro* potency, being ~4–16-fold more active against *Enterococcus* species than linezolid (Koulenti et al. 2020). Contezolid (MRX-I), a next-generation oxazolidinone with an improved safety profile, has shown potent *in vitro* activity against VRE and favorable tolerability compared to linezolid (Li et al. 2014). Its intravenous prodrug, contezolid acefosamil (MRX-4), is currently in clinical development to enable both oral and parenteral administration for the treatment of multidrug-resistant Gram-positive infections (Koulenti et al. 2020). Teixobactin, a first-in-class antibiotic that targets conserved cell wall precursors lipid II and III, has also demonstrated potent *in vitro* activity against VRE. To improve synthetic accessibility and pharmacokinetic properties, multiple analogues—such as d-Arg_4_-Leu_10_-teixobactin—have been developed, retaining strong antienterococcal efficacy and minimal cytotoxicity in preclinical studies (Parmar et al. 2018). However, as *E. faecalis* has high intrinsic tolerance to teixobactin (Darnell et al. 2019), this antibiotic may be of limited usefulness for the treatment of enterococcal infections. Additionally, MGB-BP-3, a novel minor-groove DNA binder, exerts its bactericidal activity by selectively targeting AT-rich regions in the DNA of Gram-positive bacteria. It has demonstrated potent *in vitro* activity against both vancomycin-susceptible and -resistant *Enterococcus* and is being explored for its unique mechanism that bypasses classical resistance pathways (Hind et al. 2022). While most of these candidates remain in early-phase investigation, they represent a shift away from structural analogues of existing drugs toward truly novel pharmacophores (Koulenti et al. 2020). Their continued advancement, in combination with genomic surveillance of resistance determinants, may offer a sustainable route to addressing the persistent challenge of multidrug-resistant *Enterococcus*.

Although significant progress has been made with the development of new antibiotic candidates, the issue of multidrug resistance in *Enterococcus*, especially *E. faecium*, remains a pressing challenge. This pathogen not only evades new treatments but also rapidly accumulates resistance genes, necessitating alternative approaches beyond traditional antibiotics. In response to this growing threat, phage therapy has emerged as a promising solution. By leveraging bacteriophages to target specific bacterial strains, phage therapy represents a tailored and highly adaptable complement or alternative to conventional antimicrobial therapies (Strathdee et al. 2023). For example, a clinical case study described the successful use of intravenous phage therapy, in combination with antibiotic therapy, in a 1-year-old liver transplant recipient with a life-threatening abdominal infection caused by vancomycin-resistant *E. faecium*. Treatment with a personalized two-phage cocktail led to rapid clinical improvement, and sequencing revealed that the infection shifted to a vancomycin-susceptible strain, highlighting phage therapy’s potential to both control infection and modulate resistance dynamics (Paul et al. 2021). Another case study, in which phages were used, again in combination with antibiotics, to treat *E. faecium* bacteraemia had a less favorable outcome as bacteraemia recurred after phage therapy (Green et al. 2023). In addition to phage therapy, immunotherapeutic strategies, particularly monoclonal antibodies, are gaining attention as targeted interventions against multidrug-resistant *Enterococcus*. Preclinical studies have demonstrated that monoclonal antibodies directed against *E. faecalis* surface antigens, such as aggregation substance and capsular polysaccharides, can enhance opsonophagocytic killing and confer protection in animal infection models (Romero-Saavedra et al. 2015). Notably, experimental studies have highlighted the critical role of surface-associated virulence factors, such as the endocarditis- and biofilm-associated pilus (Ebp) and gelatinase (GelE), in the pathogenesis of *E. faecalis* endocarditis. Disruption of these factors in animal models significantly attenuated bacterial virulence, reduced cardiac colonization, and altered immune responses, underscoring their potential as targets for future immunotherapeutic interventions (Nappi 2024). No licensed vaccines are currently available against enterococci, and for *E. faecium* no candidates are currently in active clinical development; nevertheless, the preclinical pipeline has expanded (Frost et al. 2023). A range of antigens, including pili (Ebp/EbpA), surface adhesins (SagA), and metal-binding lipoproteins (EfaA/AdcA/AdcAII), as well as antigens derived from membrane vesicles (Kalfopoulou and Huebner 2020) can serve as vaccine targets. Studies in murine models show protection with several of these antigens, supporting their potential as vaccine candidates (Romero-Saavedra et al. 2015, Wagner et al. 2023, Kramarska et al. 2024, Lam et al. 2024). Notably, immunization with *E. faecium* AdcA provides protective immunity to infection with other Gram-positive pathogens. This observation spurred the development of a hyperthermostable, multipresenting antigen, termed Sc(EH)_3_, which has potential to be used in vaccines against *Enterococcus, Staphylococcus*, and *Streptococcus* infections (Kramarska et al. 2024). These immunotherapies may offer a promising adjunct or alternative to conventional antibiotics, particularly in immunocompromised patients or when rapid pathogen clearance is critical. As resistance mechanisms continue to erode the efficacy of small-molecule antibiotics, immunotherapies may play an increasingly important role in the management of enterococcal infections, warranting further clinical development and translational investment.

While the rapid accumulation of antibiotic resistance mechanisms in enterococci is a cause for significant concern, there are ample opportunities for the development of future treatment modalities and innovative therapies to successfully treat infections caused by multidrug-resistant strains. The development and application of such strategies demand stringent antimicrobial stewardship and continuous monitoring of resistance patterns to effectively manage and mitigate risks associated with multidrug-resistant enterococci.
